# Improvement of Expression of α6 and β1 Integrins by the Co-culture of Adult Mouse Spermatogonial Stem Cells with SIM Mouse Embryonic Fibroblast Cells (STO) and Growth Factors

**Published:** 2013-02

**Authors:** Tayebeh Rastegar, Mohammad Bagher Minaee, Mehryar Habibi Roudkenar, Iraj Raghardi Kashani, Fardin Amidi, Farid Abolhasani, Mohammad Barbarestani

**Affiliations:** 1 Department of Anatomy Sciences, Faculty of Medicine, Tehran University of Medical Sciences, Tehran, Iran; 2 Research Center, Iran’s Blood Transfusion Organization (IBTO), Tehran, Iran

**Keywords:** SSCs, STO cells, Growth factors, α6 and β1 integrins

## Abstract

***Objective(s):*** Spermatogonial Stem Cells (SSCs) maintain spermatogenesis throughout the life of the male. Because of the small number of SSCs in adult, enriching and culturing them is a crucial step prior to differentiation or transplantation. Maintenance of SSCs and transplantation or induction of in vitro spermio-genesis may provide a therapeutic strategy to treat male infertility. This study investigated the enrichment and proliferation of SSCs co-cultured with STO cells in the presence or absence of growth factors.

***Materials and Methods:*** Spermatogonial populations were enriched from the testis of 4-6 week-old male mice by MACS according to the expression of a specific marker, Thy-1. Isolated SSCs were cultured in the presence or absence of growth factors (GDNF, GFRα1 and EGF) on STO or gelatin-coated dishes for a week. Subsequently, the authors evaluated the effects of growth factors and STO on SSCs colonization by alkaline phosphates (AP) activity and flow cytometery of α6 and β1 integrins.

***Results:*** SSCs co-cultured with STO cells and growth factors developed colonization and AP activity as well as expression of α6 and β1 integrins (*P*≤0/05).

***Conclusion:*** Our present SSC-STO co-culture provides conditions that may allow efficient maintenance and proliferation of SSCs for the treatment of male infertility.

## Introduction

The spermatogonial stem cells (SSCs) are the foundation of spermatogenesis. They can self-renew and create a large number of differentiated germ cells *in vivo *(1). A balance between SSC renewal and differentiation in adult testis is indispensable for spermatogenesis and fertility (2). The limited number of spermatogonial stem cells in an adult testis makes identification of a specific SSC marker and direct *in vivo* analysis of spermatogonial stem cells more difficult. The testis of an adult mouse contains 20,000–35,000 spermatogonial stem cells, representing 0.02-0.03% of the total cells in the testis (3). Therefore, enrichment and *in vitro *culture of SSCs provides one practical approach to investigating autologous testicular transplantation or genetic manipulation (4). Furthermore, modulating culture conditions for self-renewal, differentiation of SSCs, generation functional gametes *in vitro *and development of new therapeutic methods for infertility will be attainable (5) however difficult for a number of reasons: there is a remarkable decline in the viability of the cultured cells within one week and the cell proliferation rate is usually low, minimal or absent under adult-like culture conditions and the identification of a specific spermatogonial stem cell marker has been found challenging (6, 7). Ideally, the culture system would initiate with spermatogonia but to date, this has been a tricky endeavor and a culture system supporting propagation and differentiation of adult mouse spermatogonia is still very difficult to attain (8).

We co-cultured SSC with STO in the presence of growth factors to replicate conditions somewhat similar to the natural niche. Efficient preparation of SSCs had been characterized by histological staining and the expression of the specific germ cell markers. Our cell culture conditions produced more SSC cells *in vitro*, thereby provided more insight into maturation conditions useful for the treatment of male infertility. 

## Materiasl and Methods

*Experimental animals*

Testis cells were obtained from 4-6 week-old male mice NMRI (National Medical Research Institute). They were maintained under standard conditions with free access to the food and water. The ethics committee of Tehran University of Medical sciences approved the animal experiments, in accordance with the university guidelines.

*SSCs enrichment*

In order to obtain the testicular cells, we used a modified method published by Guan (2009). Briefly, a decapsulated testis was cut into small pieces and seminiferous tubules were transferred to Collagenase type IV (1 mg/ml) (Sigma), DNase I (10 μg/ml) (Sigma) solution. Cells were incubated at 37˚C in a 5% CO2 incubator and dispersed by pipetting every 2–5 min until the tubules separated following about 20 min. Cells were washed twice with 10 ml of phosphate-buffered saline (PBS) (Sigma) as we reported earlier (9). The dissociated testis cells suspension was overlaid on 30% (v/v) Percoll (Sigma) prepared in PBS containing 1% fetal bovine serum (FBS) (Gibco) and centrifuged at 600 g for 8 min at 4˚C. For magnetic activated cell sorting (MACS) Miltenyi Biotec protocol was assigned (Miltenyi Biotec). Sedimented cells (bottom fraction) of the Percoll gradient (3–8×106 cells in 90 μl of PBS) were incubated with 10 μl of anti-Thy-1 antibody (30-H12; Miltenyi Biotec) microbeads for 20 min at 4˚C. Following rinsing with PBS containing 0.5% bovine serum albumin (BSA)(Sigma), Thy-1^+^cells were designated by passing them through a large separating column (Miltenyi Biotec) and placed in a magnetic field (10).

SSCs culture

Enriched SSCs were cultured (6–10 ×104 cells/cm2) in a minimum essential medium-alpha (MEMα)(Gibco) containing 10% FBS, 1 x nonessential amino acids (Invitrogen), 0.1 mM 2-mercaptoethanol )Sigma), 10^3^U/ml human recombinant leukemia inhibitory factor (LIF)(B&D), 0.4 mM Pyrovate (Sigma), 1x Glutamine (Gibco), 100u/ml Penicillin and 100 μg/ml Streptomycin (Sigma), on STO (mitotically inactivated SIM mouse embryo derived Thioguanine and Ouabain resistant) or gelatin-coated dishes in presence or absence of these growth factors; GDNF (Glial Cell line-derived Neurotrophic Factor) 100 ng/ml, GFRα (Rat Glial cell line-derived Neurotrophic Factor Receptor) 300 ng/ml, (both from B&D) and EGF (Epidermal Growth Factor) 20 ng/ml (B&D) for a one week. The culture medium was changed every other day. All culture media were maintained at 32˚C in an atmosphere humidified with 5% CO2 (9). SSCs colonies were stained with Alkaline Phosphatase (Sigma) following 7 days (11). SSCs number was determined with a hemacytometer. Trypan blue (0.4%) (Sigma) whereas exclusion assays were used to determine the percentage of surviving cells following isolation and culture (12).

STO cells inactivation

STO cells were obtained from the Pasture Institute of Iran. Following the treatment of feeder cell lines (STO) with mitomycin C (10 µg/ml for 3 hr (Sigma) at 37˚C and 5% CO2), feeder cells were dissociated (0.05% trypsin EDTA) (Gibco) and cultured on gelatin coated dishes at 0.5 ×10^4 ^cells/cm^2^ for 6 hr at 37˚ C. Isolated SSCs were seeded at a density of 4×10^4^ cells/cm^2 ^(13).

Cytochemical demonstration of alkaline phosphatase 

In order to evaluate SSCs colony formation following a week of cultivation, cultured germ cells were stained with Alkaline Phosphatase. Briefly, following fixation with 10% formaldehyde (Sigma) for 10 min at 4˚C and washing with 0.2 M Tris buffer (pH 8.9), incubation with 0.01% Naphtol-AS-MX Phosphate and 0.06% Fast Violet salt (both from Sigma) in 0.1 M Tris buffer (pH 8.9 for 30 min at 37˚C, cells were washed with distilled water and observed under inverted microscope for a red bright color indicating the expression of Alkaline Phosphatase on SSCs colonies (11).

Flow cytometery 

Flow cytometric analysis (Becton Dickinson) was performed on populations of testis cells before, following enrichment and cultivation using a modified method of Shinohara (1999). Briefly, 10^6^ cells were suspended in 0.1 ml of PBS/1% FBS and 10μl fluorescence isothiocyanate (FITC) conjugated hamster anti-rat β1-integrin (CD29) antibody was added to the cells for 20 min at 4˚C. The cells were washed twice in 1 mL of PBS/FBS. Subsequently 0.1 ml of PBS/1% FBS and 10μl Phycoerythrin (PE) conjugated rat anti-human a6-integrin (CD49f) (R&D) was complemented to the cells for 20 min at 4˚C. The cells were washed twice with 1 mL of PBS/FBS. Control cells were not treated with an antibody (5).

Statistical analysis

The results were expressed as mean ± SD. The statistical significance between the mean values was determined by one-way analysis of variance (ANOVA). A level of *P≤*0.05 was considered as significant value.

**Figure 1:**
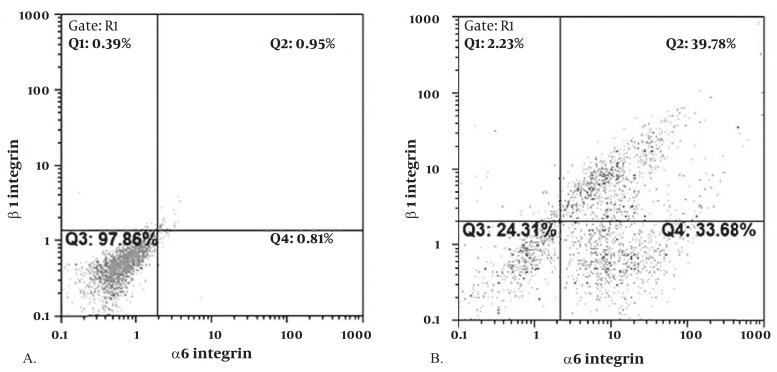
Flow cytometric analysis for detection of α6 and β1-integrin in SSCs. While there are no cells with expression of β1 and α6 integrins prior to MACS (A), there are considerable cells with positive β1 and α6 integrins following MACS purification( B)

## Results

Spermatogonial stem cells enrichment

The surface antigenic phenotype of SSCs in the mouse testis was reported to be α6-integrin, β_1_-integrin, Thy-1(CD90), CD24, GFRα_1_, Ep-CAM, GPR_125_ (14). In the current study, SSCs were isolated using anti-Thy-1 antibody and MACS. The purity of the isolated cells was determined by flow cytometery using α_6_ and β_1_-integrin antibodies. The flow cytometric analysis revealed that of the enriched Thy-1 purified cells, 67.2±2.2% (n=6) expressed β_1_ integrin, 37.7±1.8% (n=6) expressed α_6 _integrin and 30.8±1.9% (n=6) of Thy-1-enriched cells expressed both integrins (Figure 1), consistent with former data (51.3±11.9 for β_1_ integrin ) (5).

SSCs culture

**Figure 2:**
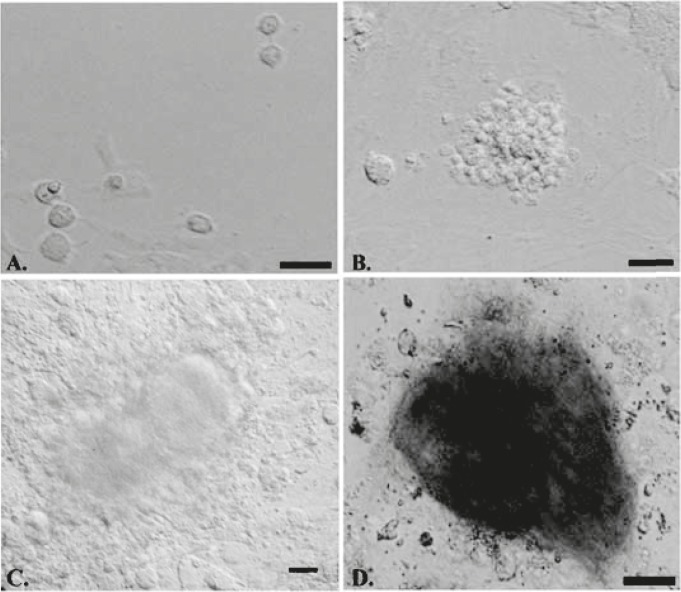
Microscopy morphology of SSCs derived from 4-6 week-old male mice in different groups. SSCs were single on the first day of cell culture (A). Following 2-3 days of cell culture, small SSCs colonies were formed (B), on the seventh day of cell culture the size of colonies was increased significantly (C) and were positive for alkaline Phosphatase activity (D)

Isolated Thy1+ SSCs were cultured on STO or gelatin-coated dishes in the presence or absence of growth factors. On the first day of culture, SSCs were single and attached to STO or gelatin coated dishes (Figure 2A). Following two days, the majority of round cells aggregated into clumps in which no cytoplasmic bridges could be discerned (Figure 2B), and then colonies were formed on days 2-3 (Figure 2C). These cell colonies were positive for alkaline phosphatase activity (Figure 2D) indicating these colonies were SSCs (10, 11).

Flow cytometric analysis

**Figure 3:**
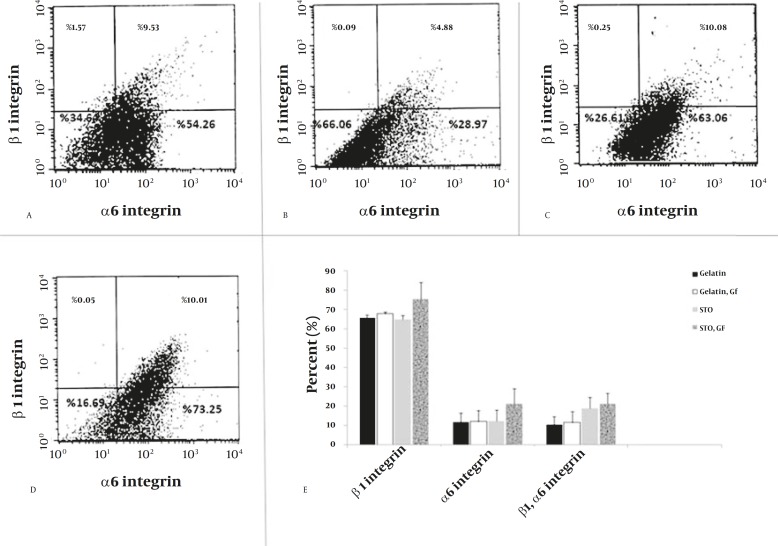
Flow cytometric analysis for the propagation of SSCs. Expression of α6 and β1-integrin in cultured SSCs on gelatin coated dishes absent growth factors (A), gelatin coated dishes with growth factors (GDNF, GFRa1, and EGF) (B), STO coated dishes without growth factors (C), STO coated dishes with growth factors (D). Comparison expression of α6 and β1-integrin in cultured SSCs in a different culture medium (E). (P≤0.05)

Following cultivating SSCs in a week, an expression of α_6 _and β_1_-integrin was determined by flow cytometric analysis. The flow cytometric analysis revealed that expression of β_1_ integrin which was up to 64% and α6 integrin up to 11% and both integrins were up to 9% on gelatin coated dishes without growth factors (Figure 3A). On gelatin coated dishes with growth factors, β_1_ integrin was up to 33% and α6 integrin up to 5%. Both integrins were up to 4% (Figure 3B). When SSCs were cultivated on STO coated dishes, β_1_ integrin was up to 70% and α_6_ integrin was up to 10% whereas β_1_, α_6_ integrins were up to 10% (Figure 3C). On STO coated dishes with growth factors, β_1_ integrin was up to 80% and α6 integrin up to 10% and both integrins were up to 10% (Figure 3D). Results in different samples in different groups (n=6/ group) were an expression of β_1_, α_6_ integrins on STO coated dishes with growth factors more than other groups (*P≤*0/05) (Figure 3E).

Our findings strongly suggest that STO cells have substantial role in propagation of SSCs and adding growth factors resulted in the improvement of SSCs propagation and expression β_1_, α_6_ integrins.

## Discussion

The isolation and proliferation of SSCs may provide a valuable tool for the treatment of male infertility. Our study employed a simple but versatile method to efficiently propagate SSCs using STO cells co-culture and supplements which comprised known important factors for germ cell survival and development. 

Mouse SSCs were enriched using anti-Thy-1 antibody and MACS technology. Our results revealed that the isolated SSCs had high purity and expressed two specific surface markers for mouse spermatogonial stem cells, for instance α_6_ and β_1_-integrin (5). Similar to our findings, Kubota *et al* (2004) used Thy-1 microbeads to isolate SSCs by MACS. However, as a source for SSCs, they used cryptorchid adult, pup, and neonate testis, in which SSCs are presented at a considerably higher percentage of total testis cells relative the amount of SSCs found in the adult testis (10). In contrast, our study used adult mouse testis as a source of SSCs, and approximately the same purity of SSCs was obtained as determined by the expression of β_1_ and α_6_-integrins (70% compared to 51%) (5) by flow cytometery, indicating the efficiency of our enrichment method. 

Because of the limited number of spermatogonial stem cells in an adult testis, ex vivo propagation of SSCs is a crucial step for *in vivo* or *in vitro *evaluations. This study investigated the propagation of SSCs co-culture with STO cells in the presence or absence of growth factors, deliberately designed to mimic the natural niche of SSCs. Our *in vitro *results strongly revealed that STO cells combined with growth factors can improve the numbers of mouse SSCs.

Feeder layers of STO cells have a beneficial role in SSC maintenance, cultivation and proliferation (9, 15). STO cells provided an environment for supporting several types of stem cells and germ cells survived for four months on a feeder layer of STO cells (16, 17). In another study, under the best conditions, 10% to 20% of the SSCs survived for a week or longer using STO feeder layers (4). In our study, we used adult mouse testis as a source of SSCs that are very low but we showed STO cells enhanced SSCs propagation and colony formation and expression of β_1_ and α_6_ integrins.

The addition of growth factors or other factors that are normally in testis to the culture medium caused an increase in the number of SSCs (6, 18, 19). Growth factors support *in vitro *expansion of SSCs and have a positive influence on SSC proliferation (20). GDNF is an important factor in maintaining the undifferentiated population of spermatogonia in the mouse testis and enhanced spermatogonial numbers and is a crucial regulator of stem cell self-renewal *in vivo* and an increased total number of these cells *in vitro* (5, 21-24). Therefore, GDNF is important factor for proliferating SSCs. Moreover, undifferentiated spermatogonia express the receptor for GDNF, and consists of the GDNF-family receptor α1 (GFRα1) and c-Ret receptor tyrosine kinase. Adding soluble GFRα1 molecules potentiate the stimulatory signal through the c-Ret receptor or modulate the signaling pathways (21, 25, 26) .Thus, the combination of GDNF and its receptor resulted in a natural condition for SSCs cultivation (20) and was used in tandem in the present study.

Alternatively, the receptor of EGF is functional in spermatogonia and EGF has been proposed to inhibit testicular germ cell differentiation. EGF also caused the proliferation of type A spermatogonia in adult rat seminiferous tubules *in vitro *(27) and might have an important role in the paracrine regulation of spermatogenesis (19). Likewise, an average concentration of EGF in blood plasma is significantly lower in infertile males. Thus EGF has an important role in the culture of SSCs.

Kubota used growth factors for culturing and replicating of SSCs (10, 20) and we used a combination of growth factors as well. But that study used neonate and pup mice that are rich with SSCs initially whereas our mice were in the adulthood stage. Our data was shown that growth factors have important role in propagating of SSCs *in vitro *but may be not enough for this purpose (as also indicated in the results of Kubota) (20). However, they obtained these results from neonate and pup mice and yet again, our results were from adult mice.

We concluded that using feeder cells and growth factors together mirrored the natural condition in testis and the observed expression β_1_ and α_6_ integrins yielded more than when they were used them separately (β_1_ integrin was up to 75% and α_6_ integrin up to 21% and both integrins were up to 21%). Feeder cells and growth factors together caused the propagation of SSCs and more expressions of β1 and α_6_ integrins. 

## Conclusion

Our findings indicated that STO feeder cells and growth factors improved SSC expansion and expression of b_1_ and a_6_-integrins. Our simple co-culture system may be applicable prior to transplantation or differentiation, although the efficiency of these cells for ART remains unknown and warrants further complementarystudy.
